# A New Health Care Paradigm: The Power of Digital Health and E-Patients

**DOI:** 10.1016/j.mcpdig.2023.04.005

**Published:** 2023-06-10

**Authors:** Andrew M. Nguyen, Alessandra Maisielou Rivera, Lisa Gualtieri

**Affiliations:** Department of Public Health and Community Medicine, Tufts University School of Medicine, Boston, MA

## Abstract

The integration of digital health into the field of medicine has seen a marked increase with the advancement of technology. Before the pandemic, a marked portion of the adult population, more than 66%, owned smartphones, and approximately 15% owned wearables. The widespread use of such devices, along with the effect of COVID-19 pandemic, has led to a transformation in health care culture that prioritizes cocreation, codesign, and collaboration. This shift promotes a model of health care centered on patient empowerment and self-management. In a recent interview with Dave deBronkart, known as e-Patient Dave, we revisited the possibilities of this new approach aimed at empowering, engaging, and equipping e-patients in the context of the pandemic. This interview with deBronkart was originally used for a graduate course on digital health. However, after noticing many reoccurring themes throughout the discussion, we decided to further explore this matter. It was discovered that participatory medicine is a new paradigm in health care, which challenges the conventional, paternalistic model and emphasizes the importance of a collaborative relationship between patients and providers. The realization of the full potential of health care can be achieved by promoting patient engagement and activation through the adoption of participatory medicine.

Despite the increasing influence of digital health on the culture of health care, comprehensive explorations into its effect and implications remain limited.^1^ This has led to digital health being an overlooked topic and a gap in our current understanding of modern medicine and health care. To address this need, we reexamine this topic through an interview with Dave deBronkart, Chief Patient Officer and Patient Empowerment Keynote Speaker, known as e-Patient Dave. Together, we delve into the ongoing discussion surrounding digital health as a cultural change, particularly in the context of the COVID-19 pandemic.

Our discussion with e-Patient Dave is split into 6 key aspects of digital health as a cultural change. We start by reevaluating the current health care models, delve into the changes that are taking place, and end by exploring the opportunities these changes may bring. Throughout this discussion, we highlight key quotes from the interview, which encapsulate each section.

By addressing these main points, we hope to highlight the importance of transitioning away from a paternalistic approach to medicine and move toward a participatory model that allows health care providers and patients the space to collaborate and cocreate on one’s individual health goals. The paternalistic model is unsustainable and leads to poorer patient outcomes and a greater increase in provider burnout. Participatory medicine can mitigate these issues and help health care reach its full potential by advancing personalized care and increasing patient involvement.

## Methods

The interview was recorded and conducted through Zoom. Questions discussed during the conversation were provided beforehand. After the recording, the authors obtained a transcript of the discussion and selected 6 key takeaways to address. The criteria used to select the main key points were on the basis of class discussions in a graduate-level course on digital health and personal experiences from each author at Tufts University School of Medicine. Each section is on the basis of a direct quote from the conversation with a combination of literature reviews, personal observations, and suggestions for improvement.

### Section 1: “To the Best of Our Knowledge”

In the traditional medical model, patients are expected to place their trust in the expertise of their doctors, accepting medical advice without question. However, this approach has faced challenges owing to rapidly evolving technologies and the need for a cultural shift toward digital health.

The COVID-19 pandemic has highlighted the importance of digital health and its potential role in transforming the health care landscape.^2^ Embracing digital health requires a cultural change that prioritizes patient engagement, collaboration, and empowerment, thereby fostering a more effective and satisfying health care experience for both patients and providers.

Although the integration of new technologies, such as electronic health records (EHRs), has shown promise in improving patient care, it has also brought about challenges, such as alert fatigue among health care teams.^3^ The constant flow of alerts, some of which are useful and others inappropriate, can lead to stress and negatively affect the quality of care for patients and job satisfaction for providers. This underscores the need for a more balanced approach to the use of technology in health care and highlights the importance of cultural adaptation in the digital health era.

The current health care paradigm is putting immense strain on the system, with the added stressors of the COVID-19 pandemic and the integration of new technologies, such as EHRs. This is leading to rising burnout rates among health care professionals and negatively affecting the quality of care for patients.^4^ To address these challenges, there is a need to reevaluate the existing paradigm and explore new ways of transforming care through digital health and cultural change. This will involve identifying the gaps in our knowledge and understanding the cultural changes needed to pave the way for a more efficient and satisfying health care system for both providers and patients.

### Section 2: “A Cultural Awakening That More Is Possible”

Digital health is the use of digital technologies for health purposes.^5^ This fast-growing field holds the promise of delivering accessible and fair care through various methods such as telehealth, public health surveillance, personalized medicine, self-tracking, wearables, genomics, and information systems.^6^ Research in this area has largely focused on evaluating its efficacy, accessibility, safety, efficiency, patient-centeredness, and equity.^7^

However, digital health is more than just technology. It is also a cultural shift in health care practices, policies, and partnerships. Adopting the definition of cultural transformation, we see it as follows:

“The cultural transformation of how disruptive technologies that provide digital and objective data accessible to both caregivers and patients leads to an equal level doctor-patient relationship with shared decision-making and the democratization of care.”^8^

What if we could design a health care system that empowers patients to be their own advocates, working in collaboration with health care providers, instead of just being passive recipients? By empowering, equipping, and engaging patients to take ownership of their own care, health care providers can move from a manager role to a mentor role, guiding patients to make informed decisions about their care. This new relationship can lead to a personalized, patient-centered approach to health care, ensuring that individuals receive the care they deserve.

Just as the saying goes, “It takes a village to raise a child,” it takes a whole health care team to provide care to a patient. Achieving this model of patient empowerment requires a cultural change.

### Section 3: “Can We Alter the Course of Care?”

The COVID-19 pandemic has resulted in a rapid expansion of digital health, providing a unique chance to change the way health care is delivered. The advancements in standards, cultural shifts, and policies provide a new outlook on the future of care that empowers patients through increased access to digital tools for personalized care and self-management.

Mobile health solutions are more accessible than ever before, with 66% of US adults owning a smartphone and 15% owning a wearable before the pandemic. In 2019, there were 325,000 health applications available.^9^ Today, mobile health applications have become a common aspect of health care solutions, advancing patient empowerment by giving them access to personalized care tools tailored to their needs.^10^ These applications can bridge social health gaps and offer constant monitoring and instantaneous health updates. Simultaneously, attention is being paid to health literacy as a social determinant of health, with efforts underway to address it.^11^ The recognition of the knowledge and skills required to effectively use mobile health tools is leading to increased digital health access and education, affecting not just the way we age, grow and live but also the way we approach health care.^12^ These tools allow patients to better understand their health conditions and make informed decisions, further promoting patient empowerment.

The backbone of the digital health revolution is common standards that facilitate communication between devices. The recent implementation of Health Level-7 Fast Healthcare Interoperability Resources 1 (HL7 FHIR) standards has enabled mobile patient health records to exchange data with EHRs.^13^ These protocols standardize and integrate data, allowing for near real-time communication between patients and providers through the internet-of-things. A survey on organizations implementing SMART/HL7 bulk FHIR data access API tools found that many organizations have already adopted these tools ahead of the 2022 deadline set by the Office of the National Coordinator (ONC) for Health Information Technology.^14^ With digital health technologies integrated with EHRs, patients can now have a more active role in managing their health data, working with their providers to cocreate their health records. This improved data exchange empowers patients to be more involved in their care and fosters personalized care plans.

In addition to standards, cultural and legal changes are also driving new care models. Under the “quantified self” movement, patients who self-track their wellness through digital health tools have gained more control and access to information.^15^ This shift has led to a disruption of the traditional doctor-patient relationship, with remote mental health services using artificial intelligence and big data analytics to proactively address symptoms during the COVID-19 pandemic.^16^ Digital phenotyping is also calling for patients to be key stakeholders in personalized care, monitoring, and interventions.^17^ In March 2020, the ONC released rules in the 21st Century Cures Act to prevent information blocking and reinforce patient rights to access and manage their health data.^18^ The American Health Information Management Association has further reinforced this focus with its Information Governance Principles for Healthcare document on information governance.^19^ These legal changes promote patient empowerment by ensuring that individuals can access, manage, and contribute to their health information, enabling a more personalized and patient-centered approach to care.

### Section 4: “We Can Perform Better if We Are Informed Better”

The COVID-19 pandemic has accelerated the shift toward a patient-centered approach in health care. Digital contract tracing played a crucial role in preventing disease transmission, and patients became active participants in public health initiatives by using their mobile devices to co-opt health data.^20^ This crisis exposed the limitations of traditional health surveillance paradigms and the need for patients to be central stakeholders in disease management and health improvement. Digital health monitoring systems provided granular information to empower patients to take on more responsibilities in their own care.

On the provider side, a similar approach has been taken with the goal of collaborating and empowering patients to play an active role in their own care. Innovative techniques, such as gamification and incentives, were adopted to address chronic disease management, and patient perspectives became integral in driving a positive health behavior. Machine-learning algorithms leveraged real-time health data from patient wearables to predict SARS-CoV-2 infection and risk, highlighting the importance of patient data in the health care process. The pandemic era has introduced new opportunities for patients to become cocreators rather than just consumers of care, challenging the traditional paternalistic model and leading to a health care paradigm that is centered around the patient.^21^^,^^22^

### Section 5: “Let Patients Help Health Care Achieve Its Potential”

Medicine is a multidisciplinary field that requires good communication, collaboration, and cooperation. But how do we achieve this? How can we help patients help themselves? What is our role in this change?

We must recognize the limitations of traditional medicine, which puts the burden of solving problems on individuals instead of the systems that cause them. Health care providers are facing burnout, mental health issues, and declining job satisfaction.^3^ These symptoms should not be seen as a personal failure but as a sign of an unsustainable health care model that is putting too much pressure on an overworked workforce. The rapid pace of change in medicine only highlights this issue because today’s practices and knowledge may quickly become outdated. Providers are faced with the difficult task of balancing their role as learners, teachers, and caregivers while adapting to constantly changing health care policy and research, particularly during the pandemic. Instead of relying on absolutes, we should embrace the iterative nature of “to the best of our ability” and “to the best of our knowledge” in health care discussions.

A new model of health care, which emphasizes cocreation and collaboration is needed to reduce provider burnout and increase patient satisfaction. To achieve this model, there needs to be a willingness from providers, patients, and patient advocates to enter into an “alliance” or pact aimed at the common good. Although this model seems to have an optimistic approach, assuming all stakeholders are in agreement, there are major limitations that can hinder the growth of this approach. Issues include patient health literacy, the complexities of navigating the health care system, and the lack on interoperability among different EHR systems.

Today, billions of people have access to a wealth of health information and communication through digital technologies.^10^ Patients have more opportunities to be involved in their own care and can bring unique perspectives that can drive new fields such as precision medicine, big data, and genomics.^23^ To make the most of this potential, providers must view patients as capable and trustworthy partners in care. Patients, in turn, must not underestimate their own ability to make a difference. This requires ongoing dialog, monitoring, and feedback among all stakeholders.

To support patient empowerment, we should invest in patient health literacy and digital health literacy. Digital health devices play a key role in enabling patients to access and understand health information.^9^ This relationship allows individuals to take control of their own health promotion, autonomy, and decision-making.^24^ However, some vulnerable populations may face barriers to accessing digital health technologies, particularly if they have limited health literacy. For example, racial and ethnic minorities are less likely to use digital health technologies than non-Hispanic Whites.^25^ The digital divide can also exacerbate disparities on the basis of socioeconomic status, education, and health literacy.^26^ Improving digital health literacy, such as health literacy, should be a public health priority that involves advancing health education and patient education.^27^

Simplifying medical information can also help overcome the complexity of health care. We can look to other industries for inspiration.^28^ For example, financial applications such as Mint, Quicken, and Acorn integrate data from various sources and simplify personal finance with data-driven insights, personalized budgets, and easy-to-use dashboards. Patients should also be able to easily manage their health data, but this is often not the case. Their records may be fragmented or incorrect, causing frustration, loss of continuity of care, and obstacles to cocreation. Instead, patients should be able to access their complete health record and make informed decisions. Although OpenNotes provides access to provider notes, only 56% of the patients can fully understand them.^29^ Medical terminology and acronyms can also make communication difficult, particularly without accessible dashboards. To truly empower patients, health care should provide personalized insights, recommendations, and care plans tailored to each person’s unique needs and goals.

To create seamless digital health experiences, software development, standards, and legal policies should continue to focus on interoperability. The inability of EHR platforms to effectively communicate with each other negatively affects the continuity of care. Although HL7 FHIR holds promise as a platform for capturing and sharing data between mobile devices, integrating it with existing systems and technologies remains a challenge. Most HL7 FHIR research is still in its early stages, focusing on prototypes and proof-of-concepts.^30^ In addition, the usability of these systems from both clinical and patient perspectives is not well understood and requires further real-world assessment through qualitative and quantitative methods. Issues surrounding data governance between data generators, data processors, and data users also need to be addressed.^31^ The rapid exchange of sensitive health information between various stakeholders also raises concerns about patient privacy, security, and authentication.^13^ To address these risks, standards and governance frameworks must be put in place.

Finally, we should embrace the structural changes to health care brought about by empowered, engaged, and informed “e-patients.”^32^ In this new participatory model, medical knowledge, skills, and attitudes must evolve. Patient knowledge and skills should be embraced and valued, and clinician attitudes should reflect the valuable contribution patients make to care. In the digital era, patients are equipped to play an active role in all 3 domains of the participatory paradigm. Updating clinician training and education models to take advantage of this is a key step in accepting this transformative shift.

### Section 6: “An Empowered Partnership Is What We Call Participatory Medicine”

Patients and health care providers are forming an empowered partnership, where patients are equipped and engaged to take part in their own care. This participatory medicine promises to unlock the full potential of health care by creating a culture shift toward personalized, patient-centered care. Personalized care considers social determinants of health, which often hinder patients from fully accomplishing their personal health goals. However, the patient-centered aspect ensures that the best interests of the patients are considered when assessing a plan of action.

With the use of more digital health tools in the practice of medicine, providers and patients, alike, are provided more informed care. Hence, patients have become cocreators of their own care, working alongside providers to achieve their goals. This patient-centered approach to health care is a 2-way street, with patients responsible for defining their own needs and providers responsible for guiding them. It is an equal responsibility between both parties—an empowered partnership.

Although an empowered partnership is ideal, it is important to also acknowledge the possibility of codestruction if cocreation and collaboration are misused. For example, a patient’s goal is to manage their chronic pain using opioids. A provider prescribes the medication but does not properly follow up with the patient or keep close monitoring. The patient does not follow up with their provider and continues to take the opioid because it has improved the pain. The likelihood of this patient becoming dependent on the medication and experiencing withdrawal symptoms is greater. The empowered partnership has become a transactional relationship. Therefore, it is important to ensure empowered partnerships are not only goal oriented but also compliant with safety and regulatory requirements.

## Conclusion

This is the present and the future of participatory medicine: unlocking the full potential of health care by empowering passive patients to become engaged patients. Although we already possess the technology and advancements to make this possible, we still have a remarkable growth to achieve in aligning societal norms with a proactive and engaged approach to medicine. The cultural change toward digital health can be realized through a multifaceted approach that includes promoting health literacy, simplifying medical information, enhancing interoperability, and embracing the evolving roles of patients and clinicians. This transformation holds the potential to revolutionize health care, improve patient outcomes, and drive a more equitable, patient-centered system. Our hope is that we all become patients unafraid to question, challenge, and seek answers.

### Limitations

Additional research, such as longitudinal studies, may be necessary to better evaluate the effect of patient engagement on health outcomes. Variables such as age, race, and sex were not discussed in the interview, nor further researched throughout this study. In addition, we acknowledge that the participatory medicine model may not be as effective in specialized care settings and suggest comparing patient and provider satisfaction in various care environments through a cross-sectional study.

## Potential Competing Interests

The authors report no competing interests.
